# Neuroimaging of Brain Networks and Function

**DOI:** 10.1155/2015/509141

**Published:** 2015-01-01

**Authors:** Hengyi Rao, Danny Jiongjiong Wang, Yihong Yang, Yong He

**Affiliations:** ^1^Center of Functional Neuroimaging, Department of Neurology, Perelman School of Medicine, University of Pennsylvania, Philadelphia, PA 19104, USA; ^2^Department of Neurology, Ahmanson-Lovelace Brain Mapping Center, University of California, Los Angeles, Los Angeles, CA 90095, USA; ^3^Magnetic Resonance Imaging and Spectroscopy Section, National Institute on Drug Abuse, Baltimore, MD 21224, USA; ^4^National Key Laboratory of Cognitive Neuroscience and Learning and IDG/McGovern Institute for Brain Research, Beijing Normal University, Beijing 100875, China

The human brain consists of multiple interconnected large scale networks. Recent advances in structural and functional neuroimaging techniques have introduced new concepts and approaches to noninvasively assess brain networks, which has attracted enormous research interest. To examine human brain function, traditional neuroimaging research typically focuses on localizing neural responses to varying external stimuli or task demands. However, task-induced phasic neuronal responses account for less than 5% of the brain's energy budget, while the majority of the brain's energy is used for intrinsic spontaneous brain activities. Although the number of human brain imaging studies and the knowledge obtained from these studies are growing exponentially, there are many more unanswered questions regarding the structural organization and the function of brain networks. In this special issue, we have invited a few papers that studied structural and functional brain networks using various neuroimaging methods including resting-state fMRI, diffusion tensor imaging (DTI), arterial spin labeling (ASL) perfusion MRI, functional near-infrared spectroscopy (fNIRS), magnetoencephalogram (MEG), and imaging genetics. These studies involve healthy populations as well as multiple clinical populations, including patients with cirrhosis, mild cognitive impairment (MCI) and Alzheimer's disease (AD), attention deficit hyperactivity disorder (ADHD), and epilepsy.

This specific issue includes several studies focusing on clinical populations. In one study by G. Zheng and colleagues, resting-state fMRI was used to examine functional connectivity changes in the progression of minimal hepatic encephalopathy and found continuously impaired intra- and intermodular connectivity in patients with cirrhosis. In another study, Y.-Y. Wai and colleagues used tract-based spatial statistics of DTI to characterize white matter integrity in different subtypes of MCI, including amnestic MCI, dysexecutive MCI, AD, and healthy controls. They found that amnestic MCI and AD patients share significant white matter damage, whereas dysexecutive MCI patients appear to have a distinct pathogenesis. In the third study, A. dos Santos Siqueira and colleagues used graph theory and analyzed a resting-state fMRI data set from healthy children and ADHD patients in the ADHD-200 database. Distinct patterns of network dysfunction were evident for both inattentive and combined ADHD subtypes. However, the authors observed that the classification scores for discriminating between ADHD and healthy subjects were close to chance and that functional connectivity estimations were strongly dependent on the sample characteristics. In the final study, H. Zhu and colleagues used resting-state MEG to identify the epileptogenic and other abnormal regions in patients with left temporal lobe epilepsy (TLE) and provided further evidence supporting the notion that TLE is not a focal focus but a multifocus disease.

A few other studies in this specific issue examined brain networks and function in normal population. In one study by H. Lei and colleagues, resting-state fMRI was integrated with genetics to examine the effect of genetic variation of the* MAOA* gene on resting brain activity in healthy male adolescents. Those with the low-activity MAOA genotype exhibited lower amplitude of low-frequency fluctuation (ALFF) in the pons compared to those with the high-activity MAOA genotype. Moreover, there was a significant genotype by ALFF interaction effect on impulsivity scores. In another study, H. Niu and colleagues examined the effects of learning on resting brain activity using fNIRS and demonstrated that 5 days of visual perceptual learning significantly altered resting oxygenated hemoglobin functional connectivity between visual cortex and frontal/central areas. In the study by C. Xu and colleagues, the regional homogeneity (ReHo) approach was used as an index in the resting-state fMRI to investigate gender differences in spontaneous brain activity among a large sample of young healthy adults. This study found significant ReHo differences in multiple cortical regions and resting-state networks between male and female subjects.

This specific issue also includes two studies on neuroimaging methods and one review paper. In one study, X. Li and colleagues compared FAIR and PICORE sequences and systematically optimized ASL imaging parameters for measuring cerebellum grey matter and white matter perfusion. In another study, M. Gorges and colleagues demonstrated a potential framework for multiparametric functional connectivity mapping through combination of resting-state fMRI with DTI. Finally, in the review paper, T. K. Das and colleagues highlighted the structure-function relationship of the brain networks using the Ising model and graph theory.

In summary, studies published in this specific issue demonstrate a few examples of continuous efforts to integrate structural and functional neuroimaging methods as well as other research modalities in order to elucidate brain networks and their function in both normal healthy population and clinical patients.

*Hengyi Rao* *Danny Jiongjiong Wang* *Yihong Yang* *Yong He*

